# Rapid Neurological Decline in a Patient With Creutzfeldt-Jakob Disease: A Case Report

**DOI:** 10.7759/cureus.87930

**Published:** 2025-07-14

**Authors:** Mishal K Siddiqui, Muhammad Y Nawaz, Haniya K Siddiqui, Tamara Williams, Brooke Williams

**Affiliations:** 1 Pediatrics, Campbell University Jerry M. Wallace School of Osteopathic Medicine, Lillington, USA; 2 Internal Medicine, Campbell University Jerry M. Wallace School of Osteopathic Medicine, Lillington, USA; 3 Neuroscience, The University of Texas at Austin, Austin, USA; 4 Biology, North Carolina Agricultural and Technical State University, Greensboro, USA; 5 Internal Medicine, Novant Health Forsyth Medical Center, Winston-Salem, USA

**Keywords:** creutzfeldt-jakob disease, neurological diseases, pakistani patients, prion diseases, south asian

## Abstract

Creutzfeldt-Jakob disease (CJD) is a rare, rapidly progressive spongiform encephalopathy caused by the accumulation of misfolded prion proteins, which undergo a transformation from the normal alpha-helix configuration (PrPC) to abnormal beta-pleated sheets (PrPSc). The disease typically leads to a rapidly progressive decline in motor, neurologic, and functional abilities, often culminating in severe disability or death within months. However, the rate of progression can vary significantly among patients, as well as the classification of CJD; being either sporadic, genetic, or acquired (infectious) with some cases demonstrating an exceptionally accelerated course.

We present the case of a 59-year-old woman with a one-month history of mood changes, irritability, temper tantrums, and progressive motor dysfunction. Neurological examination revealed basic orientation, flexed upper extremities with dyskinetic movements of upper and lower extremities, and a prominent startle reflex. Over the next two weeks, while admitted, her condition deteriorated rapidly, resulting in complete incapacitation and inability to respond within a total of six weeks. MRI and EEG findings were highly suggestive of CJD, and the diagnosis was ultimately confirmed through cerebrospinal fluid (CSF) analysis.

This patient’s rapid neurological decline within a short time frame is atypical even within the spectrum of CJD cases. Factors influencing disease progression include age of onset, comorbidities, specific CJD classification, and possibly even the pathogenicity of the misfolded prion proteins. The acceleration seen in this case raises important questions about unidentified biological or environmental factors that could influence disease trajectory. While CJD is universally fatal, recognizing and characterizing these rapidly progressive forms can refine diagnostic criteria and enhance early supportive interventions.

This case highlights the importance of early diagnostic imaging and CSF testing in patients presenting with unexplained neuropsychiatric and motor symptoms. Furthermore, it underscores the need for clinicians to recognize atypical and accelerated presentations of CJD, including fluctuating neurological signs. Although early detection cannot alter the disease course, it may allow for improved quality of life and prevention of disease transmission in those with the genetic subtype. Awareness of these variations in disease progression can ultimately help guide clinical decision-making and future research into neurodegenerative disorders. This case also highlights the importance of equitable healthcare for minority populations. As a South Asian woman with a rare disease, our patient faced potential barriers to timely diagnosis and specialized care. This underscores the need for culturally competent medicine and advocacy to ensure all patients receive dignified and comprehensive care.

## Introduction

Creutzfeldt-Jakob disease (CJD) is a prion disease characterized by rapidly progressive dementia, myoclonus, ataxia, and motor dysfunction. Prions are abnormal infectious proteins that misfold and induce the misfolding of normal cellular prion proteins, ultimately leading to neurodegeneration. The normal proteins are found on cell surfaces, but the misfolding causes their deposition intracellularly and extracellularly in a cascade effect, leading to multiple protein aggregates. Over time, these protein clusters spread throughout the brain, resulting in plaques and spongiform changes that result in widespread neuronal loss and brain deterioration. While prion transmission can occur through ingestion of contaminated meat or exposure to infected medical instruments, most cases of CJD arise sporadically without a clear source of infection [1].

CJD typically affects individuals between the ages of 40 and 75, with peak onset occurring between 60 and 65 years old [1]. The disease commonly presents with progressive memory loss, but additional symptoms such as insomnia, visual disturbances, depression, and muscle weakness may also occur. Once symptoms begin, the disease progresses rapidly, with a median survival of 6-12 months. Most patients experience a steady decline over four to six months, leading to complete cognitive and motor incapacitation before death [2].

In this case, a woman exhibited an exceptionally rapid decline, experiencing profound cognitive and motor deterioration within just six weeks, far faster than the expected four- to six-month progression. This unusually aggressive course raises the possibility of a more virulent prion strain contributing to the accelerated deterioration. Rapidly progressing CJD complicates timely recognition and intervention, underscoring the importance of early detection through advanced imaging techniques such as magnetic resonance imaging (MRI) and cerebrospinal fluid (CSF) biomarker analysis. Identifying factors that contributed to this unusually fast disease progression may provide insight into the underlying mechanisms driving aggressive forms of CJD.

## Case presentation

Patient overview

The patient is a 59-year-old woman with a past medical history of obstructive sleep apnea, hypothyroidism, late-onset type I diabetes, and ST-elevation myocardial infarction with drug-eluting stents placed in the left anterior descending (LAD) and right coronary artery (RCA), presented to the emergency department (ED) for the fourth time in four weeks with multiple brand new symptoms she had never experienced before.

Presenting symptoms

The patient presented to the ED with an inability to provide history due to significant weakness and altered mental status. According to family members, the patient had initially developed insomnia, right-sided hearing loss, numbness, and tingling on the right side of the body, and a myocardial infarction - all occurring two months prior to the current presentation. These changes were then attributed to her cardiac event.

One month prior to the current presentation, the patient had returned to baseline, maintaining full functionality, engaging in daily activities, caring for grandchildren, and even dancing well at a wedding. However, she subsequently developed progressive right-sided numbness and tingling, cramping in the right hand and foot during sleep, and an unstable gait with right foot dragging. Additional symptoms included altered taste and smell, decreased appetite, nausea, vomiting, central chest pain, and a significant unintentional weight loss of approximately 30-40 pounds. Behavioral changes such as mood swings and temper tantrums were also noted. By the time of presentation, the patient had experienced complete right-sided hearing loss, with progressive hearing decline on the left side.

Initial laboratory findings

On admission, vital signs were notable for a blood pressure of 113/65 mmHg, pulse rate of 82 beats per minute, temperature of 98.2°F, and a respiratory rate of 24 breaths per minute. Laboratory studies revealed a white blood cell count of 11.4 × 10^9^/L, a serum glucose level of 242 mg/dL, an aspartate aminotransferase (AST) of 47 U/L, and an alanine aminotransferase (ALT) of 46 U/L.

Neurological examination

Neurological examination demonstrated profound weakness on both sides of the body, and the patient was unable to follow or engage in conversation. She exhibited writhing, spastic, chorea-like movements in all extremities, with inward rotation of the left upper extremity and clenched fist posture, despite preserved strength across all muscle groups. A prominent startle reflex was elicited. She was oriented to person, place, and time and able to follow basic commands, though she remained significantly agitated and fearful throughout the encounter.

Diagnostic workup

First Brain MRI (Initial Presentation)

MRI of the brain with and without contrast demonstrated areas of hyperintensity on diffusion-weighted imaging (DWI) involving the basal ganglia and subcortical regions, with characteristic cortical ribboning seen in the insular and parietal areas. Notably, there was bilateral DWI hyperintensity involving the caudate nuclei and cortical abnormalities suggestive of early prion disease.

Follow-Up Brain MRI (57 Days Later)

Repeat MRI revealed progression of the previously noted abnormalities, with increased diffusion signal in the bilateral caudate nuclei, putamen, insular cortices, posterior temporal lobe cortices, and medial frontal lobes. These findings were more pronounced compared to the earlier study, further supporting a diagnosis of CJD.

**Figure 1:**
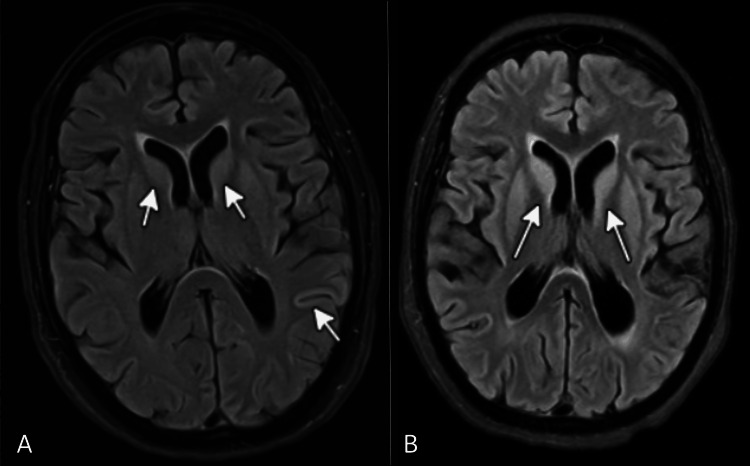
Serial Brain MRI Demonstrating Progressive Changes in Creutzfeldt-Jakob Disease (CJD) (A) Initial brain MRI showing diffusion-weighted imaging (DWI) hyperintensities involving the bilateral caudate nuclei and subcortical cortical ribboning in the insular and parietal regions, suggestive of early prion disease (white arrows). (B) Follow-up brain MRI performed 57 days later reveals the progression of DWI hyperintensities involving the bilateral caudate nuclei, putamen, insular cortices, posterior temporal lobe cortices, and medial frontal lobes, consistent with advancing CJD (white arrows).

Representative MRI images from the patient’s initial presentation and follow-up study are shown in Figure 1, illustrating the progressive nature of the observed abnormalities.

Electroencephalography (EEG)

Initial EEG demonstrated left hemispheric lateralized periodic discharges (LPDs), which later evolved into generalized periodic discharges (GPDs), consistent with encephalopathic changes seen in prion disease.

CSF Analysis

CSF appeared colorless and clear with no xanthochromia. The CSF was negative for cryptococcal antigen, autoimmune encephalopathy and paraneoplastic markers, meningitis/encephalitis panels, oligoclonal bands, and cytologic evidence of malignancy. However, additional CSF biomarkers were obtained to further evaluate for prion disease. The results demonstrated markedly elevated levels of total protein, 14-3-3 gamma protein, and T-tau protein, along with a positive real-time quaking-induced conversion (RT-QuIC) assay. These findings strongly supported the diagnosis of probable CJD. The patient’s CSF profile, summarized in Table 1, further supported the diagnosis of prion disease, with key biomarkers demonstrating marked abnormalities.

**Table 1:** Cerebrospinal Fluid (CSF) Findings in the Patient The patient's CSF showed markedly elevated red blood cells (RBC), glucose, and total protein. CSF biomarkers revealed a positive real-time quaking-induced conversion (RT-QuIC) assay, confirming the presence of prion disease. T-tau protein was elevated at 14,220 pg/mL (reference: 0-1,149 pg/mL), and 14-3-3 gamma protein was markedly elevated at 11,6801 AU/mL (reference: 173-1,999 AU/mL). The likelihood of prion disease was calculated to be greater than 98%, meeting the Centers for Disease Control and Prevention (CDC) diagnostic criteria for probable prion disease.

CSF Parameter	Patient's Values	Reference Range	Significance
RBC	47/mm^3^	0/mm^3^	Elevated
Glucose	149 mg/dL	40-70 mg/dL	Elevated
Total Protein	82 mg/dL	15-45 mg/dL	Elevated
14-3-3 Gamma	11,6801 AU/mL	73-1,999 AU/mL	Elevated
T-tau Protein	14,220 pg/mL	0-1,149 pg/mL	Elevated
RT-QuIC	Positive	Negative	Likelihood of prion disease: >98.0%, meeting CDC criteria for probable prion disease

Additional laboratory and imaging workup

Comprehensive testing ruled out heavy metal toxicity and vitamin B deficiency. Hepatic pathology was excluded via negative viral hepatitis serologies and normal ammonia levels. Computed tomography (CT) scans of the chest, abdomen, and pelvis showed no evidence of malignancy.

Clinical course

Over the first week of hospitalization, the patient developed worsening difficulty with oral intake, necessitating the placement of a nasogastric tube. Within two weeks, respiratory distress emerged, prompting transfer to the intensive care unit (ICU). Cognitive function continued to decline, and she became unable to respond to basic questions regarding personal identity or location. Although she had intermittent episodes of appearing alert, these periods did not yield any meaningful communication or cognitive engagement.

By the fourth week, her clinical trajectory showed waxing and waning, with brief improvements overshadowed by persistent deficits. A percutaneous endoscopic gastrostomy (PEG) tube was ultimately placed for nutritional support. Although she was briefly evaluated for discharge with home health, acute respiratory decompensation required the initiation of high-flow oxygen therapy.

By the seventh week, progressive hypoxemic respiratory failure necessitated intubation. At that time, the family was counseled regarding the terminal nature of the illness, and goals-of-care discussions were initiated. The patient no longer followed commands and demonstrated intermittent myoclonic jerks, which were deemed non-epileptic.

Subsequent discussions with the family centered on tracheostomy placement and transfer to a long-term acute care hospital (LTAC) versus compassionate extubation with a do-not-resuscitate/do-not-intubate (DNR/DNI) order. The family faced significant cultural and religious challenges in making decisions regarding end-of-life care. Despite trials of non-invasive ventilation via continuous positive airway pressure (CPAP), the patient remained profoundly encephalopathic and unable to protect her airway, precluding safe extubation.

At the time of the most recent assessment, the patient was tracheostomy dependent and remained sedated, intermittently opening her eyes only to noxious stimuli, with no evidence of meaningful neurological recovery. Over the course of her prolonged hospitalization, she experienced multiple complications, including extended-spectrum beta-lactamase (ESBL)-producing *Klebsiella pneumoniae* infection, *Stenotrophomonas maltophilia* tracheitis, and bilateral lower extremity deep vein thromboses (DVTs). Despite the absence of neurological improvement and the accumulation of medical complications, she remains on full-code status almost seven months after her initial presentation. This aligns with her family's wishes to pursue continued aggressive care.

## Discussion

CJD is a rare, rapidly progressive neurodegenerative disorder caused by misfolded prion proteins that induce widespread neurotoxicity. This patient presented with a one-month history of mood changes, irritability, temper tantrums, and progressively worsening motor dysfunction. Over the following two weeks, her condition deteriorated rapidly, leading to complete incapacitation and loss of verbal communication within six weeks. This trajectory is exceptionally aggressive compared to the typical course of sporadic (classic) CJD, which progresses over four to six months [3]. Given the rapid decline, absence of a family history of prion disease, and the low likelihood of variant CJD based on the patient’s lack of exposure to high-risk sources such as raw or contaminated meat, the diagnosis was presumed to be sporadic CJD. Genetic testing was not pursued after discussion with the family due to low clinical suspicion for a familial form, and the family has expressed that they are not currently interested in pursuing a posthumous autopsy.

CJD progression can be conceptualized in three stages. The initial stage presents with nonspecific symptoms such as fatigue, dizziness, decreased activity, anxiety, depression, and memory disturbances. The second stage is marked by a rapid decline in cognitive function, impacting communication, gait, and neuropsychiatric stability. This phase includes motor paralysis, aphasia, apraxia, sensory disturbances, and dystonia. Finally, the third stage results in the loss of voluntary movement, severe cognitive and physical incapacitation, and myoclonus. Many patients enter an akinetic mutism state, in which voluntary movement ceases, and meaningful verbal responses are absent. On average, this state is reached within 3.2 months after symptom onset, with mortality typically occurring within 6 to 12 months due to complications such as infection or aspiration pneumonia [4]. The extraordinarily rapid six-week decline observed in this patient is a significant deviation from the expected disease course. While CJD is known for its swift progression, this case suggests an aggressive form of the disease, potentially linked to a more virulent sporadic CJD (sCJD-MM1), familial CJD (fCJD-E200K), or MV2 subtype, each of which has been associated with accelerated neurodegeneration [5].

The diagnosis of CJD is based on a combination of clinical presentation, EEG, MRI, and CSF analysis. In this case, the EEG initially showed left hemispheric LPDs, which progressed to GPDs, a pattern commonly seen in CJD. MRI findings revealed DWI hyperintensity in the caudate nuclei, putamen, insular cortex, posterior temporal lobe cortex, and medial frontal lobes bilaterally, which is consistent with progressive neurodegeneration due to prion disease [6].

The presence of a positive RT-QuIC assay is particularly diagnostic, as this test detects misfolded prion proteins with >98% specificity according to the Centers for Disease Control and Prevention (CDC) diagnostic criteria [7]. Additionally, markedly elevated T-tau and 14-3-3 protein levels indicate rapid neuronal degeneration, further corroborating the aggressive nature of this case. Compared to similar literature, a report of a 79-year-old woman with CJD showed rapid deterioration within two months, with similar EEG, MRI, and CSF abnormalities, particularly positive 14-3-3 protein and periodic discharges on EEG [8]. However, our patient's six-week decline represents an even more extreme case, reinforcing the possibility of a highly aggressive prion strain.

The rapid progression of CJD, combined with its incurable nature, placed an immense emotional burden on the patient’s family. The sudden decline made it difficult for them to comprehend the severity of the condition, and they initially struggled to accept the irreversibility of the disease. The concept of an untreatable, degenerative disorder conflicted with their expectations of medical intervention, and cultural and religious beliefs played a significant role in shaping their perspective. The family had difficulty understanding the disease pathology and the lack of effective treatments, which led to hesitation in accepting palliative care. Their strong belief in divine intervention and hope for recovery caused initial resistance to ceasing interventions that would not alter the disease course. Studies have shown that in cultures with high religious influence, families often request continued aggressive interventions despite a poor prognosis, struggling with the emotional and spiritual implications of withdrawing care [9].

This case exemplifies an extremely aggressive form of CJD, characterized by a six-week progression from mild symptoms to complete incapacitation. The patient’s diagnostic findings, particularly elevated T-tau and 14-3-3 protein levels, a positive RT-QuIC assay, and extensive MRI hyperintensity, align with the pathophysiological markers of prion disease. Compared to similar literature, this case highlights an outlier in disease rapidity, suggesting a particularly virulent prion strain or genetic predisposition. Beyond the clinical aspects, this case underscores the profound emotional and cultural challenges families face when confronted with an incurable neurodegenerative disease. The difficulty in understanding the prognosis, accepting palliative care, and navigating religious and societal expectations necessitates a compassionate, culturally informed approach from healthcare providers. Effective patient-family counseling and tailored educational resources can help bridge the gap between medical recommendations and cultural perspectives on end-of-life care.

## Conclusions

This case illustrates an extraordinarily aggressive progression of CJD, with a six-week decline that significantly deviates from the expected disease course. The diagnostic findings, including positive RT-QuIC, elevated T-tau and 14-3-3 protein levels, and extensive MRI abnormalities, confirm the presence of prion disease and suggest an especially virulent form. Compared to similar cases in the literature, the rapid deterioration seen here highlights the variability in disease trajectory and the potential influence of prion strain heterogeneity or genetic susceptibility. Beyond the clinical implications, this case also sheds light on the emotional and cultural challenges that families face when confronted with an untreatable neurodegenerative disease. The difficulty in understanding prognosis, reluctance to discontinue interventions, and religious and societal considerations emphasize the importance of a compassionate, culturally sensitive approach to patient care. By improving patient and family education and fostering clear, empathetic communication, healthcare providers can help navigate the complex decision-making process in terminal neurodegenerative diseases like CJD.
